# Study of Histopathological Changes in the Placenta in Preeclampsia

**DOI:** 10.7759/cureus.30347

**Published:** 2022-10-16

**Authors:** Kashish Bhojwani, Anil Agrawal

**Affiliations:** 1 Pathology, Jawaharlal Nehru Medical College, Datta Meghe Institute of Medical Sciences, Wardha, IND

**Keywords:** proteinuria, preeclampsia, hypertension in pregnancy, pregnancy-associated hypertensive disorders, eclampsia

## Abstract

Pregnancy disorders include, most commonly, hypertensive disorders, which include gestational hypertension, preeclampsia, and chronic hypertension with superimposed preeclampsia. Various etiological factors prove to be both risks as well as protective factors, which include genetic factors, maternal smoking (it is inversely related to preeclampsia; that is, smoking decreases the incidence of preeclampsia), and other medical comorbidities such as hypertension, diabetes, asthma, and others, including older maternal age and high body mass index. Usually, high maternal and fetal mortality rates are seen with the diagnosis of hypertensive disorders in pregnancy, and severe morbidity is seen in cases of preeclampsia, eclampsia, and HELLP syndrome (hemolysis, elevated liver enzymes, and low platelet count), along with raised levels of liver enzymes and blood disorders such as low platelet counts. Preeclampsia is considered one of the most serious consequences of pregnancy. These disorders are often presented as newly diagnosed high blood pressure and proteinuria during the last trimester. But it can prove to be fatal for both the mother and the fetus. Though the causative factors of preeclampsia are still unknown, specific clinical and histopathological researchers propose that preeclampsia can be due to pathological changes in the placenta. The basic aim of this article is to discuss various histopathological changes in the placenta due to preeclampsia, but minor topics that affect the pathophysiology of the placenta due to preeclampsia are also mentioned. Furthermore, the effective management of maternal syndrome complications in pregnancy has also been discussed.

## Introduction and background

Preeclampsia is a complex disorder and various studies have shown genetic, environmental, immunological, and nutritional roles in its occurrence, though the exact cause largely remains debatable [1]. It is basically an abnormality in pregnancy that involves many systems and is mainly manifested by varying degrees of placental abruption accompanied by the release of placental factors in the bloodstream, which ultimately leads to high blood pressure and multi-organ failure. This majorly affects the placenta, leading to fetal growth retardation (FGR) and stillbirth. Once preeclampsia has been established as a diagnosis, it cannot be fully cured but can only be controlled to avoid complications at the time of delivery. So the dates and timings of delivery have to be planned positively to avoid unacceptable maternal and fetal outcomes. Specific clinical trials have proposed some prognostic and diagnostic methodologies that can improve maternal and fetal results. The delivery time in females diagnosed with preeclampsia in the late term of pregnancy has been noted. Different drugs are being tried and tested to control hypertension and avoid its fatal consequences during pregnancy [2]. Preeclampsia mainly targets the first pregnancy. Common clinical manifestations are high blood pressure associated with proteinuria. These manifestations are seen due to the release of placental factors in the bloodstream due to syncytiotrophoblastic complications. Two types of preeclampsia are known now: early-onset and late-onset preeclampsia. Early onset is due to defects in placental formation, while late-onset can be due to normal aging of the placenta and cardiovascular and metabolic disorders owing to the mother's genetics. Placental and maternal etiological factors vary from individual to individual. Current studies focus on the interactions of the placenta with the uterus in early pregnancy. Hence, it is aimed to implement these results to find new techniques to predict, prevent, and treat preeclampsia [3].

Placental growth factor (PGF), a protein encoded by the PGF gene, can help predict, diagnose, and treat preeclampsia. It has been found to have beneficial effects on feto-placental circulation, and it also promotes trophoblastic growth. Investigations are being conducted to discover the regulation of the expression of placental growth factors. Low levels of this growth factor in circulation are seen before the actual clinical manifestations of preeclampsia begin. Hence, low PGF levels can be a marker of abnormal placentation. PGF can be a promising marker in preeclampsia and can be used to target resources for females who are at greater risk of severe pregnancy complications. Some animal models during the experiment have been treated successfully with PGF supplemented externally. However, actual confirmation of the role of placental growth factor in pregnancy-associated hypertension requires more exploration. This shows the role of PGF in the placenta's normal and abnormal development and the management of preeclampsia [4]. Further characteristics of complicated preeclampsia include abnormally high blood pressure, protein in the urine, dysfunction of the brain and spinal cord, hepatocyte injury, low platelet counts, reduced urine output, pulmonary edema, and cerebrovascular injury with intrauterine growth retardation in the fetus. Females diagnosed with these complications should be kept under observation to confirm the disorder, evaluate the disorder's severity, check disease progression, and effectively manage the complications [5].

Preeclampsia, which is also an inflammatory disorder, involves multiple systems and has been seen as the primary etiological factor of fetal and maternal mortality over decades. Fractalkine (CX3CL1), a part of the cytokine family, plays multiple roles in the structural framework of the immune system. During the inflammatory process, fractalkine promotes tissue damage and the invasion of inflammatory cells. Some researchers aim to find out the changes in fractalkine in the cells of the placenta of pregnant females with hypertension and also how to correlate these changes with clinicopathological variables [6]. The common clinical manifestations are high blood pressure and multiple organ failure, mainly involving the kidney, lungs, and liver. Currently, the safest management line for preeclampsia is to terminate the pregnancy, followed by delivery of the fetus and placenta. In females diagnosed with preeclampsia in the early trimester of pregnancy, specific measures are required in order to manage the severity. As stated earlier, preeclampsia is also associated with cardiovascular risk later in life. So, the management of preeclampsia should be such that it has no serious impact on the fetus as well as on the mother [7]. An objective scoring system of the placenta to diagnose preeclampsia histopathologically is also mentioned in the text.

## Review

The leading obstetric disorders, which are the main etiological factors of morbidity and mortality, are eclampsia, preeclampsia, hemolysis, and elevated liver enzymes. They are often associated with both maternal and perinatal mortalities and morbidities. Hence, physicians need to make a quick, timely, and appropriate diagnosis to prevent unwanted consequences due to these syndromes. Generally, females present at 21 weeks or more of gestation and <2 days after delivery with classical manifestations of preeclampsia, which are hypertension and proteinuria [8]. Eclampsia and preeclampsia are common complications of pregnancy. These complications ultimately result in placental-maternal circulation complications. More than 39% of pregnant females fall prey to complications such as seizures due to elevated blood pressure. Hence, a differential diagnosis of preeclampsia on the basis of histopathological changes can be made. The following research aims to confirm an objective scoring system of the placenta histopathologically for a proper diagnosis of preeclampsia. Based on some studies spanning over two years, two groups were considered: 50 cases of preeclampsia/eclampsia and 50 females as controls with the normal placenta. The histopathological changes in the placenta for both groups were studied, and then a method based on scores was devised to evaluate the complications of preeclampsia disorder. In the results, 2 was the maximum score, and 0 was the minimum score for maternal side infarcts, calcification, villous membrane thickening, and fibrin deposition. The least score of 0 and the highest score of 1 were assigned to syncytial knots. A Chi-squared/Fisher’s exact test was used to analyze the relationship between the variables of the placenta histopathologically with the clinical detection of eclampsia, preeclampsia, and control [9]. An analysis of variance (ANOVA) test was used to compare the histopathological scores of preeclampsia, eclampsia, and control groups. A p-value below 0.05 was considered to be clinically significant. It was seen that an essential relation between various histopathological factors of the placenta such as fibrin deposition, maternal side infraction, calcification, villous basement membrane thickening, and syncytial knots and clinical findings of eclampsia, preeclampsia, and control groups was present. A median score of 2 correlated significantly with the normal group, whereas a median score of 4 and 6 correlated significantly with preeclampsia and eclampsia, respectively. So, from the above results, we can conclude that the newly devised system of scores can prove to be a reasonable basis for clinically establishing the diagnosis of preeclampsia and eclampsia in females along with other abnormalities related to uteroplacental insufficiency [9]. Certain pathological variations in the placenta can be seen in pregnancy-induced hypertension (PIH), resulting in decreased blood supply to the placenta.

In other research, a case-control study was carried out between July 2015 and September 2017 in which the placentas of both normal pregnancies and those complicated by preeclampsia/eclampsia during the gestation period were included. The placentae were divided into two groups: group 1 included pregnancy with hypertension, and group 2 included normal pregnancy (control). Then, as per procedure, both macroscopic and microscopic examinations were conducted on both groups of the placenta. It was found that the average weight of the placenta (464±60 g) was more in the control subjects than in the PIH group (410±60 g). This difference was clinically significant (p=0.001). The average weight of the fetoplacental mass ratio (6.18± 0.604) was higher among the PIH group than in the control group (6.05±0.501). This difference was clinically insignificant (p>0.05). Hence, we conclude that a considerable difference between the average birth weight of infants and the average weight of the placenta in the control and PIH groups was seen. Some other findings included increased syncytial knots, cytotrophoblastic cellular proliferation, villous stromal fibrosis, and fibrinoid necrosis in the placenta with preeclampsia [10].

**Figure 1:**
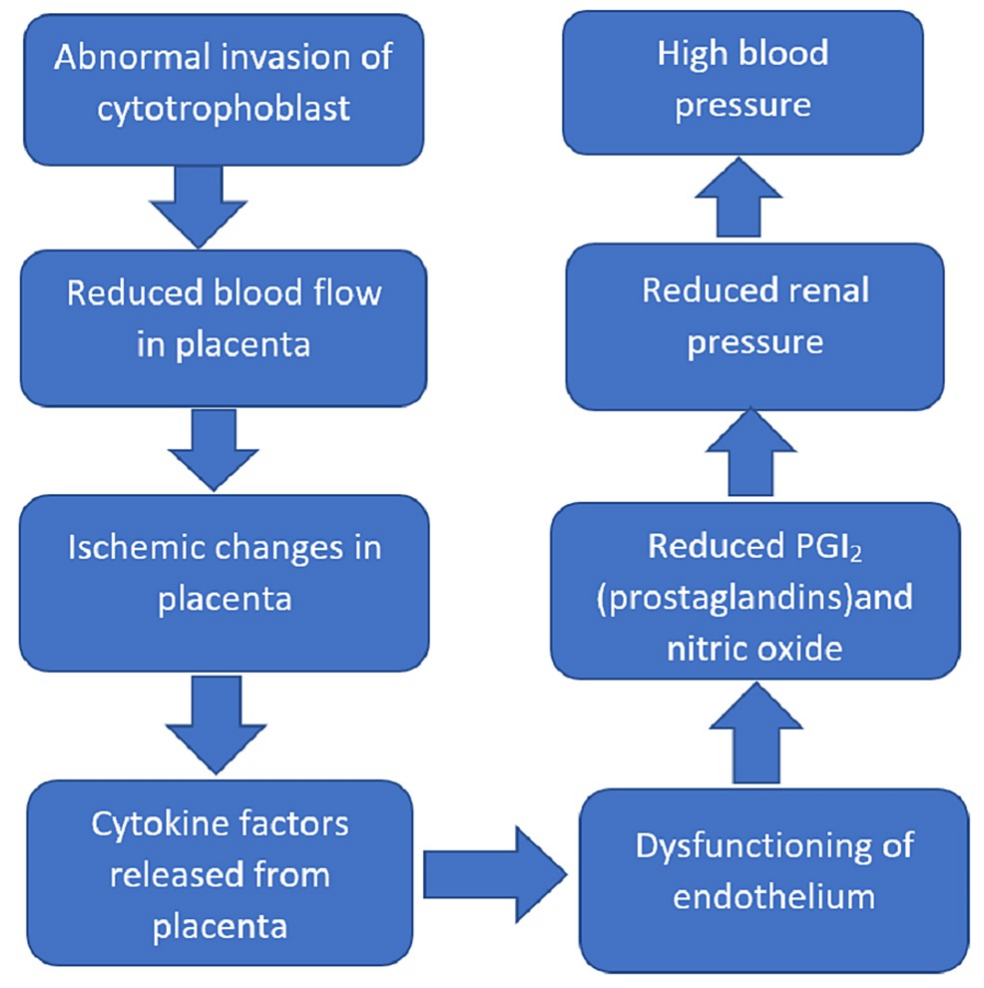
Pathogenesis of preeclampsia Self creation by author

Another consequence of gestational hypertension is FGR. In pregnancies that are complicated by FGR, many histopathological features of the placenta are found recurrently, which include villous infarction, vascular changes on the maternal side, and villous morphological changes, even though one-fourths of placentae associated with fetal growth retardation do not show any defect in the morphology on routine examination [11]. Though such changes can also affect pathologically less severe pregnancies, the positive value of these results for abnormal fetal growth retardation in a random case remains low (Figure 1). But the placental pathology shows various patterns with clinical subgroups. The combination of placental bed and parenchymal lesions in FGR with abnormal uterine artery Doppler velocimetry is similar to preterm preeclampsia (PET). Also, there is an association between FGR and abnormal Doppler results of the artery of the umbilicus, lesions of fetal stem arteries, and terminal villous hypervascularity. But it is seen that the placenta of pregnancies associated with PET or FGR, at term or 36-38 weeks of gestation, presents with less histopathological abnormality than the early diagnosis of the disorder and no signs of other biochemical profiles. Hence, various pathological findings of the placenta are seen in fetal growth retardation, which varies from morphologically indistinguishable to severe uteroplacental vasculopathy, also not a single pathological finding associated with high specificity or sensitivity. Some abnormal maternal uteroplacental perfusion features are often associated with features such as the severe early onset of FGR, which generally overlaps with the severe early onset of PET. These features are secondary to impaired extra villous trophoblastic invasion and result in fetal growth retardation, which has a late onset and usually represents a different group with less significant histopathological variations. Further research planned in the future may be aimed at assessing the collected data of histopathological studies from multiple sources into large databases with centralized pathology review and allowing the alignment of differing clinic-pathological relations and understanding of pathophysiology [11]. Also, details about the clinical and pathological characteristics of placental mesenchymal dysplasia (PMD) made severe by preeclampsia are unknown.

A 38-year-old female pregnant for the first time was referred at the 18th week of pregnancy. Ultrasonography was done, a fetus with intrauterine growth retardation was identified, and a thickened placenta with multiple cysts was observed, which suggested PMD. Then, at the 22nd week of gestation, the female developed severe high blood pressure with protein in urine and raised levels of serum soluble FMS like tyrosine kinase-1 levels. Ultimately, it was decided to terminate the pregnancy to save the patient. The weight of the placenta was 460 g, and no abnormal histopathological results of either dysplasia or proliferation were found in the trophoblast. An examination of the villous chromosome was 46 XX, karyotype. Along with the pathology of vascular endothelial dysfunction characteristic of the placenta in PE cases, enhanced expression of sflt-1 in the syncytiotrophoblast of the enlarged villi was confirmed by immunohistochemistry as a novel finding in this condition. Monitoring of the serum sflt-1 value is suggested to be a valuable predictor of the pathological change associated with extremely early severe PE in PMD [12]. Preeclampsia affects 4-6% of pregnancies and is detected initially by the twin presentations of hypertension and protein in the urine. Updated points include maternal organ injury, kidney damage, liver injury, vascular and neurological manifestations, uteroplacental dysfunction, and fetal growth retardation [5]. Large numbers of pregnancies diagnosed with preeclampsia were found to have abnormal signs in histopathological reports of the placenta, such as areas of ischemia. Though there was similarity in pathology, it was still more prominent in severe cases as compared to mild cases of preeclampsia, indicating that both severe and mild preeclampsia can have the same exact primary etiology [13].

**Figure 2:**
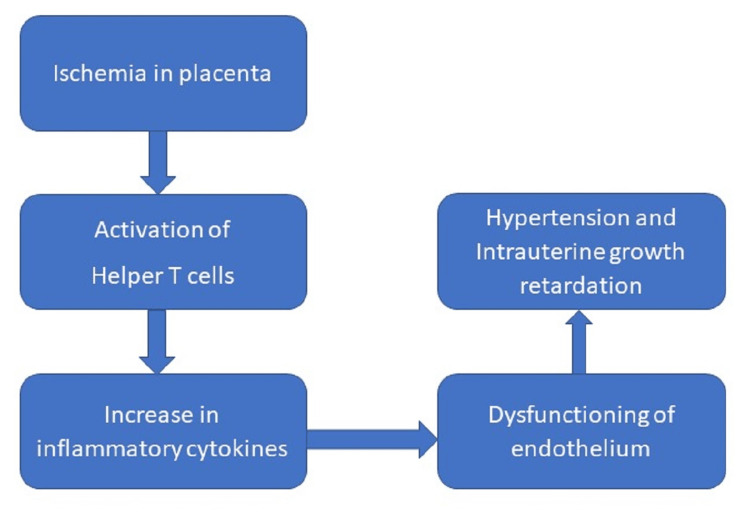
Preeclampsia due to ischemia of placenta Self creation by author

Findings of histopathology of the placenta can indicate changes due to ischemia, placental inflammation and membranes of the fetus, and other changes pointing towards fetal anemia. This can prove to be a threat to the neonate and can cause complications post-delivery or in early childhood. Chronic inflammation of the villi occurs due to a maternal rejection reaction. This reaction can cause FGR. Preeclampsia in the early term can lead to severe ischemia in the placenta. Hence, placental examination should be done if any abnormal growth is seen in the fetus or if the fetus is stillborn, if any congenital disease is detected, or if any case of infection is diagnosed [14]. Attempts to prevent eclampsia and preeclampsia have not been successful, and rates of recurrence have been high [15]. Women who become pregnant for the first time are predominantly prone to suffering from preeclampsia. This is often accompanied by high maternal and fetal mortality and morbidity. Generally, due to preeclampsia in pregnancy, the balance between oxidation, coagulation, and vasomotor activities is changed due to accelerated sensitivity to angiotensin II which is often related to decreased synthesis of prostaglandins (vasodilators), over-activation of the sympathetic nervous system, high lipid levels are often associated with increased synthesis of lipo peroxide, and improper implantation of cytotrophoblast in the spiral artery of the maternal side. This would often lead to vasoconstriction, and the worst consequence could be endothelial injury along with ischemia of both the uterus and placenta. So, clinical manifestations of the above characteristics are arterial hypertension, protein in the urine, and retention of sodium ions (Figure 2). It has also been noted that women who are more susceptible to developing preeclampsia have specific genetic polymorphisms in their genomes.

Preeclampsia is thus caused by a number of factors, as is evident from the preceding discussion. Rarely do all the factors interact, making it difficult to establish a sequence of events that leads to preeclampsia. When all risk factors interact, it might be difficult for physicians to identify a specific risk factor for a specific pregnancy, and this has been challenging for researchers as well [16]. Investigations of preeclampsia also involve histopathological examination of the placenta as it is an easy method to examine the main organ involved in the disorder. Some characteristic features of preeclampsia are vascular placental arterial dysplasia. Though they do not confirm the diagnosis since they are found in large numbers, they indicate some abnormality in the placenta. Characteristics of vascular lesions are the microscopic engagement of the basal layer known as atherosis, along with uteroplacental arterial dysplasia and its associated effects. Other features are retroplacental hematoma and infarction, including all minor insults to the placenta. The above features are usually seen in complicated cases of preeclampsia and usually indicate the development of placental IUGR [17].

The causation of preeclampsia is not completely known, despite decades of research. But any abnormality in the placenta related to immune response and malfunctioning of the placenta can initiate the development and progression of the disease [18]. Initial markers of the disorder can prove to be promising in order to understand the pathogenesis and also to develop new techniques to predict and prevent the disease eventually [19]. Though the knowledge of the mechanism of these markers has progressed, the line of management has remained unchanged for the last 50 years. One line of management includes postponing the delivery of females diagnosed with severe preeclampsia. This improves pregnancy outcomes, but not without risk to the mother [20]. Other researchers are making various efforts and also trials are being done to create a unique and powerful biochemical basis of analysis in serum to detect as well as predict preeclampsia [21]. Some recent research is trying to prove that preeclampsia is a disorder that mostly occurs due to a partial defect in the immune system of the maternal side against antigens in the fetal blood expressed on trophoblastic tissue [22].

Though no proper treatment to prevent preeclampsia has been discovered to date, it is still possible that some treatments to manage the complications need to be started in the early trimester of pregnancy so as to avoid complications in the late trimester. New investigations are being tested for this [23]. Also, the pharmacological (drug) treatment for chronic high blood pressure in pregnancy has changed over the past few years. It could be because the first-line drugs have undergone various kinds of research in the past. New studies have confirmed the efficacy and safety of other second-line drugs for the treatment of preeclampsia in pregnancy [24]. This disorder, being idiopathic, is manifested across a wide spectrum of clinical manifestations, ranging from mild signs to severe complications [25]. Though there is little success in the knowledge of the clinical spectrum and the line of management of preeclampsia, the etiology and basic pathology remain unknown [26]. Early detection of preeclampsia would allow the immediate initiation of treatment to prevent complications [27]. Hence, the pathogenesis of preeclampsia has been a popular topic for research for obstetricians [28]. Also, another common disorder of pregnancy is arterial hypertension. But preeclampsia and arterial hypertension are two different disorders with different pathogenesis and different lines of management [29]. The prevention of this disorder is limited to the absence of a perfect biological marker that is quite sensitive and also appropriately specific [30]. Apart from this, it has been found that in the case of multiple pregnancies, the last pregnancy that had complications due to preeclampsia can be a risk factor for the current pregnancy [31].

Probably the basic etiology of preeclampsia is an abnormal growth of trophoblastic cells, possibly triggered by altered maternal immunotolerance [32]. But the characteristic finding related to histopathological studies in the placenta is the narrowing and atherosis of certain branches of the uterine artery, which is present near the deciduomyometrial interface [33]. Now the role of the placenta in the causation of preeclampsia is unchallenged. Evidence such as maternal syndrome of preeclampsia mainly occurring due to maternal systemic inflammatory response (MSIR) is being evaluated [34]. The causative factors of preeclampsia are diagnosed in the early trimester of pregnancy. But from progressive ongoing research, we understand that the development of early-onset and late-onset preeclampsia along with IUGR does not compulsorily indicate a common origin of etiology [35]. In spite of many ongoing kinds of research, the pathogenesis, diagnosis, and prevention of preeclampsia remain puzzling [36]. But much current evidence indicates that there can be many underlying etiologies or predisposing factors that can lead to endothelial malfunctioning and give rise to a group of symptoms such as high blood pressure, protein in urine, and edema. These signs allow us to make a diagnosis of a syndrome called preeclampsia [37].

## Conclusions

The first and foremost goal of this article was to discuss the histopathological changes in the placenta due to preeclampsia. Certain non-classical and atypical characteristics of preeclampsia/eclampsia were also discussed. Also, systemic progression towards detection and management of cases of preeclampsia with uncommon features is being described. Also, we tried to present the current understanding of preeclampsia, focusing on fetal growth retardation, mild or severe complications due to preeclampsia, and relation of preeclampsia with cardiovascular risk factors. Hence, the earlier the disorder is diagnosed and confirmed, the better the fetal and maternal outcomes are. Thus, this is the reason why it is necessary to find out the risk factors in high-risk patients through appropriate screening methods. This will allow early detection and early management, thus ensuring better outcomes for both mother and child. To support this, the study of the histopathology involved in the placenta due to preeclampsia can be beneficial.
